# Supplementary material for: The role of Methyl-CpG binding domain 3 (Mbd3) protein in epileptogenesis
Source: PLoS One. 2025 Oct 8;20(10):e0324580. doi: 10.1371/journal.pone.0324580 (PMC12507310; doi:10.1371/journal.pone.0324580)
Supplement: Attachment — Submitted filename: Nizinska et al rebuttal.odt [file pone.0324580.s004.odt]

Dear Prof Biagini and Reviewers,
Thank you for the opportunity to revise and resubmit our manuscript PONE-D-25-22943, entitled “The role of Methyl-CpG binding domain 3 (Mbd3) protein in epileptogenesis. We sincerely appreciate the time and effort that you and the Reviewers have invested in evaluating our work. We have carefully considered all the comments and suggestions, and we have revised the manuscript accordingly.
Below, we provide a detailed response to each point raised by the Reviewers. 

In-house Editorial Comments:
Comment 1: When submitting your revision, we need you to address these additional requirements. 
Answer: Reference style has been corrected. The title page has been formatted according to instructions. Files have been named according to the instructions
Comment 2: Please provide an amended statement that declares *all* the funding or sources of support (whether external or internal to your organization) received during this study, as detailed online in our guide for authors at http://journals.plos.org/plosone/s/submit-now.  Please also include the statement “There was no additional external funding received for this study.” in your updated Funding Statement. 
Please include your amended Funding Statement within your cover letter. We will change the online submission form on your behalf.
Answer: The Funding statement has been updated and is included in the cover letter: 
“This work was supported by an OPUS grant 2015/19/B/NZ4/01401 (KL, KN). There was no additional external funding received for this study.”

Point-by-point response to ALL reviewers' comments
Reviewer #1: 
Comment: Please add the age and number of rats in the animals’ subheading of the methods section.
Answer: To address this comment, we extracted some information from the "Animal Surgery" chapter and moved it to a newly created "Animals" chapter, and added the required information to that chapter. The added sentence reads:
“Altogether, 220 animals were used; two animals died during PTZ kindling.  The body weight at the start of the experiment was 280-320 g.”

Comment: Please write in vitro in an Italic format in whole parts of the manuscript.
Answer: We apologize for this error. It has been corrected throughout the manuscript.

Comment: Did you check the normality of data? What was the relevant test/s? Please add its detail in the statistical analysis.
Answer: Normality of data distribution was assessed using the Kolmogorov–Smirnov test. This information is now added to the chapter ‘Statistical analysis”

Comment: In the results section, for describing statistical results, please provide details of the statistical test used, and full statistical reporting of the results. Full statistical reporting should include the number of observations, the test statistics (eg t, f values), exact probabilities and degrees of freedom for each of the observations where statistics are provided.
Answer: To address this comment, we have improved the description of statistics in the “Materials and Methods” section and have rewritten the whole “Results” section to provide the requested information. 

Comment: The quality of provided Figure 3 is not good and it’s blur. Please provide a better image. In addition, it’s not clear which parts of the brain were evaluated in the figure. For better clarification, please also add an image with lower magnification.
Answer: In response to this suggestion, we provide higher-quality images acquired using confocal microscopy. We have also added information on the localization of the presented neurons in the description of the Figure 3 and added relevant information to Materials and Methods section 

Reviewer #2
Comments: 1) The anayses of transcriptome data seem to be preliminary. Additional bioinformatic analyses help to highlight which enriched functional terms may be more associated with overexpression of Mbd3. 
In response to this comment we have substantially updated the manuscript. We have performed an additional functional enrichment analysis focusing on Gene Ontology Biological Process (GO BP) terms for differentially expressed genes at each analyzed time point. Additionally we have updated the analysis of Clusters, changing the analysis performed with Reactome to GO BP providing a more accessible for the reader view of biological processes potentially associated with Mbd3 overexpression. The results are now presented in the updated Table 1, with enriched GO BP terms for each time point. Furthermore, we have substantially revised and extended the Discussion section to elaborate on the biological roles of selected genes and pathways identified in the analysis, thereby providing a more comprehensive interpretation of the transcriptomic changes observed.
Comments: 2) Represented genes on transcriptic data should be analysed by qPCR both in vitro and in vivo.
3) Further, the CpG status of some represented genes may also be detected under epileptic and Mbd3 overexpression conditions, which can provide strong evidence for the role of Mbd3 in epileptogenesis.
Answer: We fully agree that validating transcriptomic data by qPCR both in vitro and in vivo, as well as investigating the CpG methylation status of selected genes under epileptic conditions and Mbd3 overexpression, would provide critical mechanistic insights into the role of Mbd3 in epileptogenesis. At this stage, we are actively seeking funding to pursue these directions, which we intend to address in a follow-up study and subsequent manuscript.

Comment: 4) Fig 1E, F and G: Related Western blot images should be present in this figure.
Answer: To address this comment, we have added to Figure 1 panel H, presenting the Western Blot image, which shows the only statistically significant difference between protein levels following PTZ injection, that is, the 4h time point in the entorhinal cortex/amygdala. 
Due to the large number of samples analyzed, the individual time points were run on separate gels. As a result, assembling a single panel with the corresponding Western blot images would require splicing individual lanes from different blots. We believe that such a figure would not be informative and could be potentially misleading. For this reason, we decided to show only representative images of the time point with a statistically significant difference.

Comment: 5) Some typo errors need to be revised.
Answer: We apologize for the typo errors. The text was checked for spelling, grammatical,  and stylistic errors.
