# Supplementary material for: The role of Methyl-CpG binding domain 3 (Mbd3) protein in epileptogenesis
Source: PLoS One. 2025 Oct 8;20(10):e0324580. doi: 10.1371/journal.pone.0324580 (PMC12507310; doi:10.1371/journal.pone.0324580)
Supplement: Attachment — Submitted filename: Nizinska_et_al_rebuttal_auresp_2.odt [file pone.0324580.s005.odt]

Dear Prof Biagini and Reviewers,
Thank you for the opportunity to revise and resubmit our manuscript PONE-D-25-22943, entitled “The role of Methyl-CpG binding domain 3 (Mbd3) protein in epileptogenesis. We sincerely appreciate the time and effort that you and the Reviewers have invested in evaluating our work. We have carefully considered all the comments and suggestions, and we have revised the manuscript accordingly.
Below, we provide a detailed response to each point raised by the Reviewers. 

Point-by-point response to ALL reviewers' comments
Reviewer #1: 
Comment: All concerns have been fully addressed. As a minor issue, please rewrite the conclusion of the abstract.
Answer: The abstract has been rewritten according to the Reviewer's suggestions and now reads:
Methyl CpG binding domain 3 (Mbd3) protein belongs to the MBD family of proteins and is responsible for reading the DNA methylation pattern. Our previous study revealed increased levels of Nucleosome Remodeling and Deacetylase (NuRD) complex proteins, including Mbd3, in the brains of epileptic animals. The present study investigated whether the Mbd3 protein level determines the seizure threshold.
We demonstrate that seizures induced by pentylenetetrazole (PTZ) cause a transient, brain area-specific increase in Mbd3 protein levels in the entorhinal cortex and amygdala. Overexpression of Mbd3 in the amygdala using AAV decreased anxiety, increased excitability in the open-field test, and accelerated epileptogenesis in the PTZ-kindling model. In vitro, mRNA profiling using RNA-seq in a model of magnesium deficiency-induced epileptiform discharges revealed complex, time- and state-specific changes in gene expression. Genes regulated by Mbd3 overexpression were associated with the Wnt and Notch pathways, potassium channel function, and GABAB receptor signaling.
Our findings indicate that increased Mbd3 expression has pro-epileptic properties and contributes to the regulation of multiple pathways potentially involved in seizure development. Significantly, seizures themselves transiently elevate Mbd3 levels, suggesting a potential vicious circle that may aggravate disease progression. Targeting the pro-epileptic effects of Mbd3 could therefore represent a novel therapeutic approach in epilepsy.
